# Automatic workflow for the classification of local DNA conformations

**DOI:** 10.1186/1471-2105-14-205

**Published:** 2013-06-25

**Authors:** Petr Čech, Jaromír Kukal, Jiří Černý, Bohdan Schneider, Daniel Svozil

**Affiliations:** 1 Laboratory of Informatics and Chemistry, ICT Prague, Technická 5, Prague 6, 166 28, Czech republic; 2 Department of Computing and Control Engineering, ICT Prague, Technická 5, Prague 6, 166 28, Czech republic; 3 Faculty of Nuclear Sciences and Physical Engineering, CTU Prague, Trojanova 13, Prague 2, 122 00, Czech republic; 4 Institute of Biotechnology AS CR, v. v. i., Vídeňská 1083, Prague 4, 142 00, Czech republic

**Keywords:** DNA, Dinucleotide conformation, Classification, Machine learning, Neural network, RBF, MLP, k-NN, Regularized regression, Cluster analysis

## Abstract

**Background:**

A growing number of crystal and NMR structures reveals a considerable structural polymorphism of DNA architecture going well beyond the usual image of a double helical molecule. DNA is highly variable with dinucleotide steps exhibiting a substantial flexibility in a sequence-dependent manner. An analysis of the conformational space of the DNA backbone and the enhancement of our understanding of the conformational dependencies in DNA are therefore important for full comprehension of DNA structural polymorphism.

**Results:**

A detailed classification of local DNA conformations based on the technique of Fourier averaging was published in our previous work. However, this procedure requires a considerable amount of manual work. To overcome this limitation we developed an automatic classification method consisting of the combination of supervised and unsupervised approaches. A proposed workflow is composed of *k*-NN method followed by a non-hierarchical single-pass clustering algorithm. We applied this workflow to analyze 816 X-ray and 664 NMR DNA structures released till February 2013. We identified and annotated six new conformers, and we assigned four of these conformers to two structurally important DNA families: guanine quadruplexes and Holliday (four-way) junctions. We also compared populations of the assigned conformers in the dataset of X-ray and NMR structures.

**Conclusions:**

In the present work we developed a machine learning workflow for the automatic classification of dinucleotide conformations. Dinucleotides with unassigned conformations can be either classified into one of already known 24 classes or they can be flagged as unclassifiable. The proposed machine learning workflow permits identification of new classes among so far unclassifiable data, and we identified and annotated six new conformations in the X-ray structures released since our previous analysis. The results illustrate the utility of machine learning approaches in the classification of local DNA conformations.

## Background

The antiparallel double helical structure of DNA and its self-recognition form the basis for the conservation and the transfer of genetic information. The model of the “canonical”B-DNA form proposed by Watson and Crick [1] has later been enriched by detailed structural data from single-crystal structures of the biologically prevailing B-form [2] and of its kin right-handed A-form [3,4]. In addition, the first DNA single crystal [5] revealed atomic details of a third major form of a DNA double helix, left-handed Z-DNA. The atomic resolution structures of B-DNA duplexes [6] revealed the existence of sequence-dependent structural deviations which provide the required specificity for DNA recognition by proteins and drugs [7]. The association of DNA with proteins is known to induce a local deformation of the B-form toward the A-form [8-13] in various protein-DNA complexes such as, e.g. high mobility group (HMG) proteins [14], *trp* repressor/operator complex [15], TATA box binding protein [16-18], HIV-1 reverse transcriptase [19], various DNA polymerases [20-23], zinc finger protein [24], hyperthermophile Sac7d protein [25], and *Eco*RV endonuclease [26-28]. Along the transition pathway between the B- and A-forms [29] various intermediate B-to-A conformations were identified [9,30-32]. The importance of conformational sub-states of the DNA backbone for protein binding to the minor groove was suggested by several analyses [13,33,34]. Besides the A-, B- and Z-forms, DNA can also adopt other biologically relevant structures, such as single-stranded hairpins [35], triple helices [36], three- and four-way junctions [37,38], four-stranded G-quadruplexes [39] or parallel helices [40]. Their existence indicates that DNA structure is much more polymorphic than it might be deduced from the misleading simplicity of the canonical B-DNA duplex.

The base morphology in a DNA double helix is commonly described [12,41-46] by parameters giving mutual position between bases in a base-pair (e.g., propeller twist or stagger) and in a base-step (e.g. rise or twist) [47]. The same parameters can also be used for other unusual DNA structures such as triple helices [48-50], G-quadruplexes [51] or three- and four-way junctions [52,53]. In addition, for the last two groups of structures additional specific parameters such as the G-quartet planarity [54] or the angle between the junction arms [55] were also defined. Another set of quantitative measures that can be used to characterize secondary structure of DNA are backbone torsional angles *α*, *β*, *γ*, *δ*, *ϵ*, *ζ* together with the glycosidic torsion *χ*[56]. Though the relationship between the phosphodiester backbone states and local distortions of DNA double helix was described in the '80 and '90s [57,58], the backbone was regarded as a passive link holding bases at their positions in several early analyses [7,59,60]. However, nowadays it is clear that the backbone must be considered as an active dynamic element while defining the conformational properties of double-helical DNA [34,61-69]. The main role of the backbone is in restricting the conformational space available for the placement of bases, and in steric coupling of the adjacent base steps [61]. An overall conformational flexibility of DNA thus results from the interplay between the optimal base positions and the preferred conformations of the sugar-phosphate backbone. An increasing number and quality of DNA structures led to several detailed analyses of the conformational space of the DNA backbone, most of these studies have been based on crystal structures [32,70-73] but structures determined by various solution-based techniques of NMR spectroscopy have also contributed significantly to our understanding of biology of nucleic acids [74-76]. NMR methods were successfully applied to study a dynamics of DNA phosphodiester backbone in solution [77-82], NMR studies also provide evidence for the BII states in solution and help to unravel a role of the phosphorus atom in a BI-BII transition [68,83-87].

To uncover a potential role of the sugar-phosphate backbone in the DNA structural polymorphism we have analyzed a set of carefully selected double-helical structures of naked and protein bound DNA resolved at high resolution (≤1.9 Å) [32]. We have identified all the known major conformers (AI, AII, BI, BII, and ZI and ZII) as well as several minor conformations corresponding to various transitional states between the B and A forms. The investigation was based on the technique of Fourier averaging in combination with a cluster analysis applied previously on the annotation of RNA conformers [88]. The main disadvantage of the Fourier averaging approach is that it requires a considerable amount of manual work [32]. To automate this process we introduce here a machine learning workflow that deals with two following tasks:

1. Classify data points into one of the existing classes.

2. Recognize data points that cannot be classified and identify new possible conformational classes.

**Figure 1:**
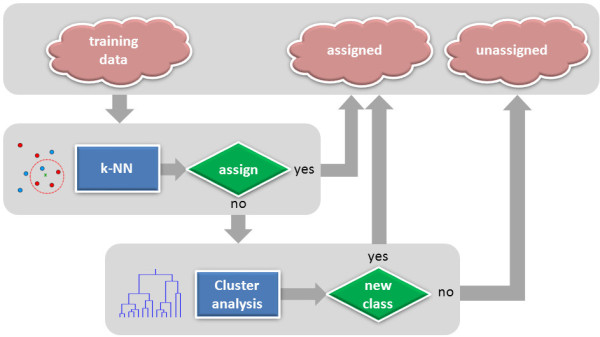
**A workflow of the classification of local DNA conformations.***k*-NN uses 11 neighbors (parameter *k*). A threshold v_crit_ = 0.001 (see explanation in the Methods section of the manuscript) was used to distinguish between data points that can be assigned to some of existing classes or cannot be assigned at all. Cluster analysis uses a modified version of the single-pass nonhierarchical *leader algorithm*[89].

The first task is accomplished by the application of the supervised machine learning approaches. In supervised algorithms a classification function is inferred from the labeled training data (i.e. each data point must be assigned to an appropriate class). As a training set we used previously published classification of DNA local conformers [32]. In the present study we applied and compared several supervised methods: multi-layer perceptron (MLP) neural network, radial basis function (RBF) neural network, *k* nearest neighbors (*k*-NN), and ridge regression (RR). The best method (*k*-NN) not only achieves high classification accuracy, but also allows identifying conformers that cannot be assigned to any of the known classes. Such conformers were subsequently investigated for the presence of new clusters using a modified clustering method based on a *leader algorithm*[89]. The proposed classification workflow (Figure 1) was applied on the analysis of X-ray data updated by structures released after 18 July 2005, and of NMR data released until 15 February 2013.

## Methods

### Data sets

For the development of the machine learning workflow we used a previousy published data set [32] consisting of 7,739 dinucleotides collected from 389 high quality crystal structures with a resolution of 1.9 Å or better and from 58 structures with unusual topologies (G-quadruplexes, i-motif, three- and four-way junctions, etc.). These structures were released into the Nucleic Acid Database [90] before 19 July 2005. In this data set we originally identified 119 conformational families. To reduce their number for the classification purposes, we critically evaluated the data for the presence of outliers and for the size and quality of the clusters. 419 outliers were removed, and the number of conformationally distinct families was condensed into 18 classes (Table 1) resulting in a data set consisting of 7,320 data points. These data were split into 4,567 dinucleotide units (*DatasetF*) classified previously by the Fourier clustering, and into 2,753 dinucleotides that were not assigned to any class in our previous work [32]. A stratified sampling was used to divide the *DatasetF* into the training (*DatasetF_train*, see Additional file 1) and test (*DatasetF_test*, see Additional file 1) sets in the ratio 80:20. *DatasetF_train* was used for classifier’s learning, and the *DatasetF_test* was used for assessing its performance. Training set contains 3,651 data points, and test set contains 906 data points. In a stratified division each of the classes is sampled with the ratio present in the total population. For example, class number 54 (BI-DNA, see Table 1) covers 42.5% of the total population, and is present in this proportion also in *DatasetF_train* and in *DatasetF_test*.

**Table 1:** Characteristics of the local B-DNA backbone conformations used in the present work

**Class ID**	**Description**	**N**	***δ***	***ϵ***	***ζ***	***α *+ 1**	***β *+ 1**	***γ *+ 1**	***δ *+ 1**	***χ***	***χ *+ 1**
8	A-DNA	325	83	205	287	294	174	54	83	199	202
13	A-DNA, BI-like *χ, χ* +1	196	89	201	275	294	162	54	89	244	244
19	A-DNA, *α*+1/*γ*+1 crank (t/t)	65	82	195	291	149	194	182	87	204	188
32	BI-to-A, O4'-endo *δ*+1	266	129	186	264	295	170	52	99	247	233
41	A-to-B, >C3'-endo *δ*, C2'-endo *δ*+1	215	90	196	280	299	179	55	142	222	256
50	BI, C1'-exo *δ*+1	392	129	181	265	300	177	50	123	246	245
54	BI	1942	136	183	259	303	181	44	138	252	259
86	BII variation in complexes	314	140	201	216	314	153	46	140	262	253
96	BII	539	143	245	170	297	141	46	141	271	257
109	BII-to-A, C3'-endo *δ*+1	20	142	213	181	297	139	52	90	273	207
110	BII-to-A, *α*+1/*γ*+1 crank (g+/t), high *β*+1	9	146	257	186	60	224	196	90	260	200
116	BI, *α*+1/*γ*+1 crank (g+/g-)	158	140	194	247	31	197	294	150	253	253
119	BI mismatches, syn/anti	11	144	189	266	303	167	53	138	70	259
121	A-to-B, >C3'-endo *δ*, anti/syn	19	100	209	278	295	174	54	128	243	67
122	BI mismatches, anti/syn, *α*+1/*γ*+1 crank (g+/g-)	8	137	196	225	33	187	295	145	257	70
123	Z-DNA, Y-R	21	147	264	76	66	186	179	95	205	61
124	Z-DNA, R-Y ZI	49	96	242	295	209	231	55	144	63	205
126	Z-DNA, R-Y ZII	18	95	187	63	169	162	44	144	58	213

**Figure 2:**
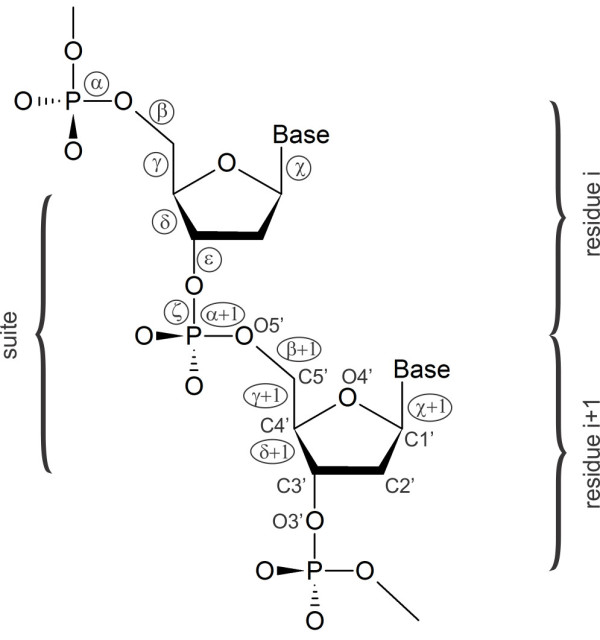
**Two repeating units in a DNA dinucleotide chain.** One residue (nucleotide) is defined from phosphate to phosphate. Conformation of each residue is given by six backbone torsion angles α, …, ζ, and by the glycosidic torsion angle χ. “Suite” goes from δ to δ+1 angles consisting from the following torsions: δ, ϵ, ζ, α+1, β+1, γ+1, δ+1.

“Class ID” is the symbolic label of the class. “Description ”is a short annotation of the class. “N” is the number of suites (dinucleotides) with the given class membership. Values of torsions represent the arithmetic means for individual classes. Torsions are defined in Figure 2.

Our machine learning classification workflow was then applied to 427 X-ray structures, resolved with a crystallographic resolution of 1.9 Å or better, and released between 18 July 2005 and 15 February 2013, which contained 8,708 dinucleotides, and to 664 NMR structures released before 15 February 2013, which contained 12,300 dinucleotides.

For our analysis a concept of a “suite” [91] was adopted. “Suite” is a conformational subset of a dinucleotide unit (Figure 2) going from sugar to sugar and consisting of 7 backbone torsions (*δ*, *ϵ*, *ζ*, *α* + 1, *β* + 1, *γ* + 1, *δ* + 1). The analysis also includes two glycosidic angles *χ* and *χ* + 1. Each data point is therefore represented by a vector composed of 9 torsion angles. In the following text we also use the convention [56] by which it is common to describe the backbone torsional angles of ~ 60° as *gauche+* (*g+*), of ~ 300° as *gauche-* (*g-*), and of ~ 180° as *trans* (*t*). For glycosidic torsion *χ* following regions are commonly used: *syn* (0° – 90°), *anti* (240° – 180°), and *low anti* (~ 200°).

### Data preprocessing

The input data (raw angle values from the 0° – 360° interval) were used either directly (in *k*-NN method) or they were normalized using one of the following methods:

1. In a geometric preprocessing each torsion was transformed from the space of dihedral angle θ ∈ {δ, ϵ, ζ, α + 1, β + 1, γ + 1, δ + 1, χ, χ + 1} to the linear metric coordinate space built up by the series of trigonometric functions {sin *nθ*, cos *nθ*} with the geometric order parameter *n* = 1, …, *D*. This preprocessing method accounts for the circular character of angular data [92,93], however it increases the length of the input vector from 9 to 2*D* × 9. This preprocessing was used in RR, MLP and RBF methods.

2. In a linear preprocessing each angle was converted into the 〈 − 1, 1〉 range. This conversion increases the performance in the Matlab environment that was used for all neural networks simulations. This preprocessing was used in MLP and RBF methods.

Depending on the classification method, the output data (i.e., the class membership of individual data points) were encoded in two different ways:

1. The original class numbering (see Table 1) was used in *k*-NN.

2. Classes were renumbered to the interval 1-18, and the class membership was then encoded as a binary vector of the length 18. This encoding was used in RR, MLP and RBF methods.

### Training and cross-validation

Each classifier is characterized by one or more parameters that are tuned to capture the underlying relationships in the training data set, and that influence the ability of an algorithm to perform accurately on new, previously unseen examples (the generalization ability). The combination of one particular method (e.g. MLP neural network) with particular values of parameters (e.g. number of hidden neurons equaling to 10) is designated as a model. The most appropriate values of the parameters were chosen using a well established method of *k*-fold cross-validation. In *k*-fold cross-validation, a training set is divided into *k* parts. A classifier is trained *k*-times, each time leaving out one of the subsets (the so-called validation set), which is used to assess the classifier’s performance. At the end, the final validation error is obtained as the average of all errors from *k* individual validation runs. In the present work a 10-fold cross-validation was adopted using the stratified division of the *DatasetF_train*. The quality of the trained model was evaluated by the Mean Squared Error of Validation *MSE*_*validation*_.

(1) $$\mathrm{MS}E_{\mathrm{validation}}{=}_{n}^{1}\Sigma_{i=1}^{n}{\left(P_{i}-T_{i}\right)}^{2}$$

where *P*_*i*_ is the predicted class membership and *T*_*i*_ is the known class membership. To smooth out possible biases caused by an unfavourable random data set division, the 10-fold cross-validation was repeated 10 times, and the final *MSE*_*validation*_ was obtained as an average of validation errors from all individual runs. A model with the lowest *MSE*_*validation*_ represents the “best” model. Once it was identified the final model was trained using the whole *DatasetF_train*. The quality of individual classifiers was compared using the *MSE*_*test*_ calculated for the *DatasetF_test*.

### Classifiers

#### A multi-layer perceptron (MLP)

MLP represents the most common architecture of neural networks. It consists of simple processing units (neurons) arranged into three or more layers: one input layer, one or more hidden layers, and one output layer. Every neuron in one layer is connected to every neuron in the following layer, and no intra-layer connections exist. The strength of neuron connections is represented by numerical weight values. The weights are free variables of the system which are determined during the training phase. Neurons transform a numerical input to an output value via the transfer function. In the present work, a two-layer perceptron consisting of one input, one hidden and one output layer was used. Several transfer functions were tested: linear, log-sigmoid and tan-sigmoid. Log-sigmoid function is given as

(2) $$\mathrm{logsig}\left(u\right)=\frac{1}{1+e^{-u}}$$

and tan-sigmoid function is given as

(3) $$\mathrm{tansig}\left(u\right)=\frac{e^{2u}-1}{e^{2u}+1}$$

where a potential *μ* of a neuron is given as $u=\underset{i}{\sum }w_{i}x_{i}-\vartheta$, $\bar{x}=\left[x_{1},\ldots ,x_{i}\right]$ is the input vector, $\bar{w}=\left[w_{1},\ldots ,w_{i}\right]$ is the weight vector, and *ϑ* is the neuron’s bias (threshold). As the neuron’s input goes from negative to positive infinity, the log-sigmoid function generates outputs between 0 and 1, and the tan-sigmoid function generates outputs between -1 and 1.

#### Radial basis function network (RBF)

RBF is also a two-layer neural network. The input layer serves only as a mediator in passing a signal to the hidden layer. While MLP is based on units which compute a non-linear function of the scalar product of the input vector and a weight vector, in RBF the activation of a hidden unit is determined by the distance between the input vector and a prototype vector. Each hidden neuron modulates the input signal by the Gaussian transfer function called radial basis function (RBF). Each RBF is characterized by two parameters: by its center (position) representing the prototype vector, and by its radius (spread). The centers and spreads are determined by the training process. When presented with the input vector $\bar{x}$, the Euclidean distance of the input from the neuron’s center is computed by the hidden neuron, and the RBF kernel function is applied to this distance. The output from the network is constructed as a weighted sum of the RBF’s outputs. The weights are also determined in the training phase. While MLP separate the classes by using hidden neurons which form hyperplanes in the input space yielding a global approximation, RBF networks model the separate class distributions by local radial basis functions.

#### k-nearest neighbor (k-NN)

In *k*-NN method objects are classified based on the class of their nearest neighbors. A new point is assigned to the majority class among the *k* nearest points. *k*-NN is a lazy algorithm meaning that there is no explicit training phase, it makes no generalization (i.e. no underlying model of the class membership is constructed), and the decision is based on the entire training data set which must be available during the prediction phase. Euclidean distance is used as a measure of the proximity of two data points. To get the Euclidean distance between two torsion angle vectors the similarity vector $\bar{s}$ must be calculated first. Its elements *s*_*i*_ are distances between individual components of compared vectors. To correctly calculate the similarity vector $\bar{s}$ the circularity of the angular data must be taken into account. The distance *s*_*i*_ between two angles *ϕ* and ψ is given as [94]

(4) $$s_{i}=180-|180-|\phi -\psi ||$$

where both *ϕ* and ψ angles are given in degrees. The Euclidean distance d is calculated as

(5) $$d=\sqrt{\underset{i}{\sum }s_{i}^{2}}$$

In *k*-NN approach, the number of nearest neighbors *k* represents the only adjustable parameter of the method. The class membership of *k* nearest neighbors was used to assign the class of the classified point. To take into account a fact that near neighbors influence the resulting class membership more than the distant ones contributions of the neighbors were weighted by 1/d^2^. The point was assigned to the class with the highest sum of weighted contributions. However, if this sum was less than a threshold v_crit_ = 0.001, the data point was declared as unclassified. The value of v_crit_ was obtained empirically and, based on our experience, optimally balances the accuracy of the method and the number of unassigned points in the dataset.

### Regularized regression (RR)

RR [95] is a standard statistical method of linear modeling and parameter identification. In RR pattern set is represented as a pair (*X*, Y^*^), where *X* is an input matrix of the size *m* × *n*, Y^*^ is an output matrix of a size *m* × *N, m* is the number patterns, *n* is the number of inputs and *N* is the number of outputs. Ridge regression penalizes the size of the regression coefficients by the penalty calculated as a weight matrix *W* = (*X*^*T*^*X* + *λI*)^-1^*X*^*T*^Y^*^ where λ ≥ 0 is a regularization parameter and *I* is an *n* × *n* identity matrix. If the matrix Y^*^ represents the class membership, the RR response is calculated as *Y* = *XW* and the *i*th pattern is assigned to the *j*th class for which the *y*_*i,j*_ element is maximal. Main advantages of RR are fast learning procedure and ability to solve ill-posed problems with a high number of possibly dependent explanatory variables. The disadvantage of RR is the linearity of the underlying model. However, the linearity limitation can be suppressed by an appropriate nonlinear preprocessing of the data.

#### Comparing classifiers

The quality of classification models is assessed by various measures based on the counts of correctly and incorrectly predicted test data [96]. Such information can be tabulated as a confusion matrix. Each row of the matrix represents the instances in the actual class, and each column represents the instances in the predicted class. To compare the performance of various classification models this matrix is usually boiled down to the single number. In the present work two such performance metrics – accuracy and κ coefficient – were utilized. Accuracy is defined as a percentage of correctly classified data points, i.e. the main diagonal in the confusion matrix is summed (this gives the number of correctly classified data points – true positives TP) and the sum is divided by the total number of observations N:

(6) $$\mathrm{accuracy}=\frac{\mathrm{TP}}{N}•100$$

The disadvantage of the accuracy is that it does not reveal if an error is evenly distributed between classes or if some classes are really bad and some really good. To include this information the κ coefficient [97] takes into account also the off-diagonal elements

(7) $$\kappa =\frac{N\times \sum_{i=1}^{n}x_{\mathrm{ii}}-\sum_{i=1}^{n}\left(x_{i+}\times x_{+i}\right)}{N^{2}-\sum_{i=1}^{N}\left(x_{i+}\times x_{+i}\right)}$$

where *n* is the number of rows in the confusion matrix, *x*_*ii*_ is the number of observations in row *i* and column *i*, *x*_*i*+_ and *x*_+*i*_ are the marginal totals of row *i* and column *i*, respectively, and *N* is the total number of observations. κ coefficient measures the improvement of classifier’s predictions over a purely random assignment to classes.

#### Cluster analysis

The main objective of clustering is to find a grouping of similar objects within a data [98]. The objects are not labeled, and cluster analysis belongs between unsupervised methods. In the present work we used a nonhierarchical single-pass method that works on the basis of a single scan of the data set. The most common single-pass algorithm is called the *leader algorithm*[89] which is simple to implement and very fast. However, its major drawback is that it is order dependent meaning that if the compounds are rearranged in a different order then the resulting clusters can be different [89]. Therefore we developed a modified *leader algorithm* which retains high speed, and is order independent. The used algorithm consists of the following steps to provide a set of clusters:

1. Set the number of existing clusters to zero.

2. For each data point (i.e., set of nine torsions characterizing a given dinucleotide) *D*_*i*_

•Start new cluster *C*_*i*_

•Calculate a neighborhood of *D*_*i*_

•Go through all data points except *D*_*i*_. Data points belonging to the neighborhood of *D*_*i*_ are appended to the cluster *C*_*i*_

3. Remove duplicated clusters getting a set of unique clusters (a unique set).

4. Repeat until the unique set is empty

•Identify the biggest cluster *B*_*i*_ in the unique set

•If the size of *B*_*i*_ is higher than predefined threshold append *B*_*i*_ to the final set of clusters

•Identify all clusters that overlap with *B*_*i*_

•Remove *B*_*i*_ and all overlapping clusters from the unique set

In point 2. a dinucleotide belongs to the neighborhood of *D*_*i*_ if its torsion deviates from *D*_*i*_ by no more than 20° for *α*, *ϵ*, *ζ*, and χ, 30° for *β*, 15° for *γ*, and 10° for δ. These intervals were selected on the empirical basis reflecting common conformational variability (“stiffness”) of the individual torsion angles. A cluster is defined by at least six points in the presented study, which gives a value of a threshold in point 4.

## Results and discussion

### Optimal parameters of the classification methods

In MLP, we determined the input preprocessing method, the number of hidden neurons and the type of transfer function by the 10-fold cross-validation. The number of hidden neurons varied between 10 and 60 with the step of 2. We performed the cross-validation with every possible combination of linear, log-sigmoid and tan-sigmoid transfer functions using either linear or geometric preprocessing. The order parameter *n* of the geometric preprocessing was cross-validated, its values varied from 1 to 10 by one. The optimal MLP model uses the geometric preprocessing with *n* = 1 (i.e. the input vector consists of 2 × = 18 components), has 22 neurons in a hidden layer, and uses log-sigmoid (Equation 2) transfer function at hidden neurons and tan-sigmoid (Equation 3) transfer function at output neurons.

In RBF, the input preprocessing method, the number of hidden neurons and the optimum spread of the Gaussians on hidden neurons were recognized using the 10-fold cross-validation. The order parameter *n* of the geometric preprocessing varied from 1 to 10 by one, the spread varied in the interval of 0.05 and 0.025 with the step of 0.01, and the number of hidden neurons varied by one between 10 and 50. The optimal RBF utilizes a geometric preprocessing with n = 1 and has 18 hidden neurons with the spread of 0.15.

In *k*-NN, the number of nearest neighbours *k* was varied between 1 and 50. Its optimum value found by 10-fold cross-validation is equal to 11.

In RR, 10-fold cross-validation was used to set the order *k* of the geometric preprocessing and the regularization parameter *λ*. The order *k* was varied between 1 and 10 by one, and the regularization parameter *λ* was set either to 0 or it was altered by factors of 10 from 10^-6^ to 10^-3^. The optimum order of the geometric preprocessing is 6 which leads to the increase of the length of the input vector from 9 to 2×6×9 = 108. The optimum regularization parameter *λ* is zero. With this regularization parameter the ridge regression is equivalent to the standard linear regression.

### Performance of the classification methods

The accuracy of individual classification methods is summarized in the Table 2 and the confusion matrices showing the class predictions given by individual classifiers are available in the Additional file 2.

**Table 2:** Quality measures (accuracy and κ coefficient) of multi-layer perceptron MLP, radial basis function network RBF, k nearest neighbors k-NN and ridge regression RR

	**MLP**	**RBF**	***k*-NN**	**RR**
*accuracy [%]*	97,35	88,41	96,58	94,92
*κ coefficient*	0,966	0,845	0,956	0,934

These were evaluated using test set (*DatasetF_test*). The MLP model uses geometric preprocessing with the order *k =* 1, has 22 hidden neurons with the log-sigmoid transfer function and output neurons use the tan-sigmoid transfer function. The best RBF model uses geometric preprocessing with the order *k =* 1, has 18 hidden neurons with the spread of 0.15. The optimal value of *k* in *k*-NN is 11. In RR, the optimal regularization parameter *λ* is zero, and the order of the geometric preprocessing expansion *k* is 6.

The best performing classifier both in terms of accuracy and κ coefficient is the multi-layer perceptron MLP followed by the *k*-nearest neighbors *k*-NN and by the ridge regression RR. MLP and *k*-NN are both non-linear classifiers, while RR represents a linear method. The penalization of the coefficients in the ridge regression is not necessary (regularization parameter _*λ*_ is zero), and the ridge regression is therefore reduced to the standard linear regression. However, RR performs similarly to nonlinear methods due to the sophisticated preprocessing method motivated by the geometrical nature of the input angular data. A careful inspection of the confusion matrices (Additional file 2) reveals that the decrease in accuracy is caused mainly by misassignment between two pairs of classes: points belonging to the class 50 (BI conformers with the second sugar at the C1‘-exo conformation, see Table 1) can be assigned to the class 54 (BI conformers, see Table 1), and points belonging to the class 32 (BI-to-A conformers with the second sugar at the O4′-endo conformation, see Table 1) can be assigned to the class 50. Classes 54 and 50 are distinguished mainly by a slight difference in the sugar pucker at both deoxyriboses (7° in *δ* and 15° in *δ +* 1, see Table 1), the conformational transition between these classes is continuous and a limited blending of the conformers can be expected. Similar behavior show also classes 50 and 32 as they differ primarily in the *δ +* 1 torsion, the difference is 24° (see Table 1).

A poor performance of RBF comes as a surprise. Reason for this behavior can be that the classification boundary in RBF is constructed in a local manner, while MLP and RR are global methods and in *k*-NN the classification boundary is not constructed explicitly. However, an RBF confusion matrix (Additional file 2) reveals that the decrease in accuracy is also caused by misassignments between classes 50 and 51 (51 misassigned points) and between classes 32 and 50 (15 misassigned points). As explained above, certain extent of the mixing of these conformers can be expected, and we can thus conclude that a lower accuracy of the RBF network is only seeming and RBF performs similarly as the other investigated methods.

Of the studied methods, *k*-NN offers one important advantage: it allows to discriminate between conformations that can be assigned to one of the pre-defined classes and between the conformations for which such a class does not exist. From this reason we propose *k*-NN as a method of choice for the classification of local conformations in nucleic acids.

### Analysis of the newly characterized conformers

#### X-ray structures

We analyzed 2,753 dinucleotides unassigned to any class in our previous work [32], and 8,708 dinucleotides from 427 X-ray structures released between 18 July 2005 and 15 February 2013. Utilizing the *k*-NN approach (with *k* = 11 and v_crit_ = 0.001) we assigned 10,510 (91%) dinucleotides to one of 18 possible (Table 1) classes. Applying a clustering procedure on remaining 951 unassigned dinucleotides representing results of incorrect refinement of the crystallographic model or yet unidentified clusters we identified 6 new conformational classes (Table 3). A data set containing all X-ray structures analyzed in the present work can be found in Additional file 1.

**Table 3:** Characteristics of the six new conformational classes found by clustering

**Class ID**	**Description**	**N**	***δ***	***ϵ***	***ζ***	***α *+ 1**	***β *+ 1**	***γ *+ 1**	***δ *+ 1**	***χ***	***χ *+ 1**
35	BI-to-A, β+1 in g+, α+1/γ+1 crank (high t/t), anti/low anti	14	136	199	288	253	73	168	87	264	187
97	BII-DNA, α+1/γ+1 crank(t/g+), anti/low anti	13	142	294	110	149	198	55	151	260	185
113	BI-DNA, ϵ/ζ in t/g+, α+1/γ+1 crank (g+/t), anti/syn	13	143	206	61	82	204	192	146	242	68
114	BI-DNA, α+1/γ+1 crank (g-/g-), high β+1, anti/syn	18	141	201	282	307	258	304	151	236	65
115	BI DNA, high *ϵ*, anti/low anti	22	140	275	280	300	189	61	148	265	208
117	BI-DNA, β+1 in g+, α+1/γ+1 crank (high t/t), anti/low anti	19	139	196	286	249	73	172	145	263	211

“Class ID” is a symbolic label of the class. “Description” is a short annotation of the class. “N” is the number of suites (dinucleotides) with the given class membership. Values of torsions represent the arithmetic means for the individual classes. Torsions are defined in Figure 2.

Four of six new conformers can be found exclusively in two functionally distinct types of non-double helical structures. Conformer 115 occurs in four-way (Holliday) junctions, and conformers 97, 113, and 114 are found in guanine quadruplexes of the *Oxytricha nova* telomere. Other two conformers (117 and 35) are found in various DNA-protein complexes. A detailed description of new conformations is given in the following paragraphs.

#### Conformations 97, 113 and 114

These conformations are found exclusively in guanine quadruplexes (G-quadruplexes) of the *O. nova* telomere. G-quadruplexes represent biologically very interesting non-canonical DNA structures [39,99]. G-rich sequences, in which G-quadruplexes often appear, are abundant in the genome, and are found e.g. in telomeric regions [100], immonugloboluline switch regions [101] or gene promoter regions [102]. G-quadruplex of *O. nova* telomere is a well-studied [103,104] example of bimolecular, antiparallel quadruplex with the sequence d(G_4_T_4_G_4_)_2_. A core structural element of G-quadruplexes are planar G-quartets (also termed a G-tetrads) that stack on top of each other. They are connected by loops of variable length and composition whose variations lead to a wide variety of topologies of G-quadruplexes.

**Figure 3:**
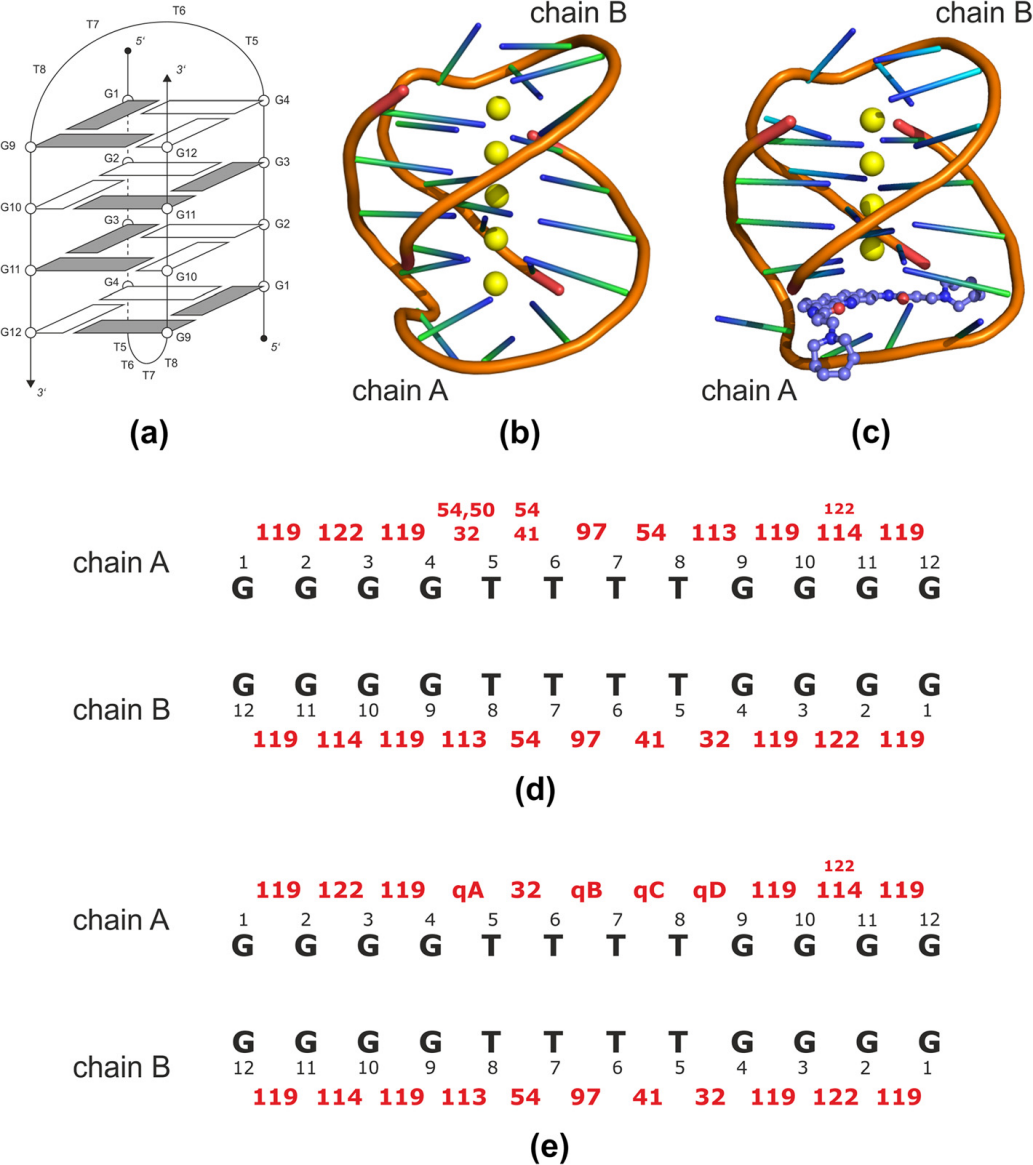
**Oxytricha nova guanine quadruplex.** (**a**) A schematic diagram of a double-stranded (bimolecular) guanine quadruplex from *Oxytricha nova* telomeric sequence (G_4_T_4_G_4_)_2_. A solid line represents a sugar-phosphate backbone. *O. nova* G-quadruplex has four G-quartets formed from nucleotides in which *syn* and *anti* conformations of the glycosidic angle alternate along each strand [105]. Shaded rectangles indicate guanine residues in syn conformation (typically χ ~ 60°-70°), clear rectangles indicate guanine residues in anti conformation (typically χ ~ 250°-260°). (**b**) A crystal structure of a bimolecular *O. nova* G-quadruplex 1JPQ [104]. Overall topology is indicated by the orange ribbon. Bases are represented by green sticks, potassium ions stabilizing the whole structure are shown as yellow spheres. (**c**) A crystal structure of a complex of *O. nova* G-quadruplex with a drug acridine 3EUM [106]. Acridine affecting the conformation of a T_4_ loop in chain A is shown in blue. (**d**) Consensus conformational map of the *O. nova* G-quadruplex. By convention, chains are numbered in the 5′-to-3′ direction. Conformational classes of individual dinucleotide steps are indicated by red numbers, their size is proportional to the frequency of their occurrence in investigated structures. A description of individual conformations is given in Tables 1 and 3. The T5T6 step adopts either a canonical BI conformation 54 if the G4T5 step is also in a canonical BI conformation, or an A-to-B conformation 41 if the G4T5 step is in a conformation 32. (**e**) Consensus conformational map of the *O. nova* G-quadruplex complexed with a drug acridine. Individual conformations shown as red numbers are characterized in Tables 1 and 3.

In our previous work [32] we were able to match several dinucleotides in *O. nova* G-quadruplex with distinct types of conformers and new conformers 97, 113, and 114 identified in the present work further enhance this structural annotation (Figure 3(a), (b) and (c)). Class 113 is a highly distorted BI-like conformation with *ϵ*/*ζ* in *t*/*g+*, *α*+1/*γ*+1 switched into *g+*/*t* values and *χ*+1 in the *syn* region (~68°). Conformer 114 represents a BI-like conformation with *anti*/*syn* arrangement of *χ* and *χ*+1 torsions, high *β* (~260°), and unusual *g-* (~300°) value for *γ*+1 torsion. Conformation 97 represents a BII conformation with *α*+1/*γ*+1 switched into *t*/*g+* values, and with *χ*+1 in *low anti* region (~185°).

By analyzing crystal (1L1H [107], 1PH4, 1PH6, 1PH8 [108], 1JB7 [103], 2HBN [109], 3EUM [106], 3NYP [110]) and NMR (156D [111], 230D [112], 1K4X [113], 2AKG [114]) structures we were able to construct a consensus conformational map (Figure 3(d) and (e)) showing the succession of conformers in the *O. nova* G-quadruplex. From the studied pool of structures, four (3EUM, 3NYP, 1L1H, and 3NZ7) represent a complex of the G-quadruplex with a drug acridine, while the rest are not complexed. Acridine binds to the quadruplex within its T_4_ loop in chain A [107] influencing a conformation of the whole T_4_ loop. Thus, we have considered naked and acridine-complexed structures separately in our analysis.

The consensus conformational map of the naked G-quadruplex is shown in Figure 3(d), and that of the complex with the acridine in Figure 3(e). Common to both are conformations present in the chain B, and in the G-tracts of the chain A. *O. nova* G-tracts exhibit a well-known 5′-*syn*-*anti*-*syn*-3′ pattern [107] of guanine glycosidic torsion angles manifested by alternating conformations 119–122–119 in the G1G2G3G4 sequence, and conformations 119–114–119 in the G9G10G11G12 sequence. The T_4_ loop in acridine complexes shows much higher conformational variability than in uncomplexed structures. This variability is manifested by the presence of unusual conformations labeled qA, qB, qC, and qD that are not homogenous enough to form distinct clusters but they do share several common structural characteristics. Conformation qA is typical by a glycosidic angle in the low *anti* (~200°) region, *β*+1 torsion in *t* (~200°) and *α*+1/*γ*+1 in *t*/*g+* combination. Conformer qB is similar to the cluster 19 (A-DNA with *α*+1/*γ*+1 crank into the *t*/*t* values, Table 1) but with a second sugar moiety in the canonical BI C2′-endo conformation. A common feature of the qC conformer is a presence of *α*+1/*γ*+1 torsions switched into the *g+*/*g+* values. qD conformation can be, based on δ and δ+1 values, labeled as BI-like with *α*+1/*γ*+1 switched to the *g-*/*t* values, *β* in *g+*, and with *χ* + 1 in the *syn* region.

Described conformational assignment demonstrates that *O. nova* G-quadruplexes are conformationally homogenous structures that could be decomposed into the clustered conformers some of which are unique to these structures (conformations 97, 113, 114, and 119). The complexation with the acridine molecule results in a higher conformational variability of the T_4_ loop compared to the G-tracts.

#### Conformation 115

**Figure 4:**
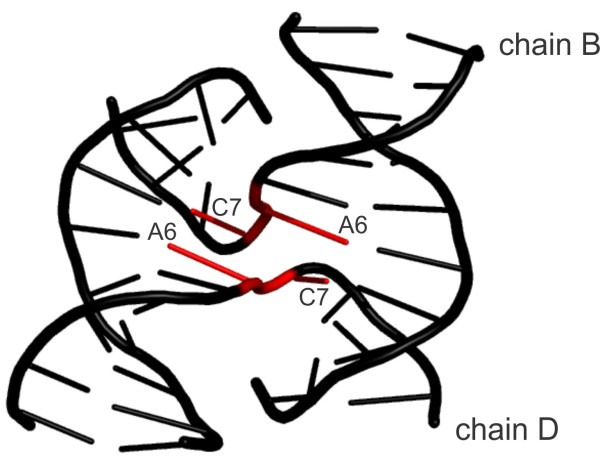
**Structure of a four-way (Holliday) junction in an inverted repeat sequence 1DCW **[115]**.** The backbone between residues A6 and C7 in chains B and D (shown in red) adopts an unusual BI-like conformation 115 with high ϵ (~ 275°) and A-like χ+1 (~ 208°).

This class describes a conformation found exclusively in Holliday (four-way) junctions. It was noticed previously [32] as potentially existing, but only the larger data set including the recent data lead to its identification. It can be characterized as a BI-like conformer with unusually high *ϵ* (~275°) and A-like *χ*+1 (~208°). This conformation is found in the sharp bend of the DNA strand between residues number 6 and number 7 (Figure 4).

#### Conformation 117

This class represents a BI-like conformation with both δ and δ+1 torsions in the C2′-*endo* region but its torsions *α*+1, *β*+1 and *γ*+1 acquire values (~250°, 73°, and 172°, respectively) not typical for the BI conformer 54. In addition, glycosidic torsion χ+1 of the second residue is in A-like *low anti* region near 210°. This conformation was almost exclusively observed in protein/DNA complexes, about a half of them in complexes of nucleosome-core particle. The DNA bending induced by interactions between DNA and histone octamer has been explained [32] by the periodic alteration of BI and BII conformers with occasional insertion of conformation 116 (Table 1). The new conformation 117 is its rarer kin found only in some nucleosome structures located outside the protein/DNA interface.

#### Conformation 35

Class 35 can be characterized as a transitional BI-to-A conformation with the first residue in BI and the second residue resembling an A-form whose character is disturbed by unusual values of *β*+1 (*g+*, ~70°), α+1 (~250°), and *γ*+1 torsions (*t*, ~168°). This conformation occurs in diverse protein/DNA complexes, about a half in DNA complexed with polymerases. Dinucleotides in this conformation are in direct contact with protein atoms via the phosphate charged oxygen.

### NMR structures

We clustered a set of 12,300 dinucleotides from 664 NMR structures released before 15 February 2013 (see Additional file 1) utilizing *k*-NN procedure with *k* = 11 and v_crit_ = 0.001. We assigned 11,313 dinucleotides (92%), and subsequently applied a new round of clustering to the remaining 987 points. However, clustering did not reveal any new conformation that would be present in NMR and not in X-ray data.

**Figure 5:**
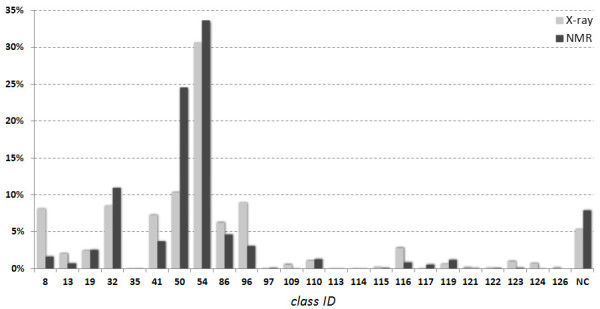
**Comparison of a fraction of individual conformational classes (Tables**1**and**3**) identified in structures resolved by X-ray (816 structures) and NMR techniques (664 structures).**

Across-the-database assignment of dinucleotide conformers for 816 X-ray and 664 NMR DNA structures exhibit similar general features (Figure 5). The BI conformer 54 is dominant in both data sets, and the BI conformer 50, the BII conformers 86 and 96, and several A-DNA conformers (8, 19, 32, 41) are also significantly populated. Similar qualitative features of the assignment of the local DNA backbone conformers demonstrate that DNA in solution and in the crystal phase, which is highly hydrated, show similar behavior. However similar the overall features are, both populations also exhibit significant differences. Perhaps the most noticeable is the difference of the overall BI population (the conformers 50+54+116) that forms 65% in NMR, and only 47% in crystal structures. The BI conformers are more populated in NMR than in crystal structures, striking is especially a large population of the conformer 50 in NMR (27%, compared to just 11% in crystals). Also the fractions of some other conformers differ significantly. NMR structures have more populated the mixed B-A conformer 32, and crystal structures the canonical A-form 8, the mixed A-B form 41, and the BII conformers 96 and 86. NMR structures have a slightly larger proportion of unassigned dinucleotides than crystal structures, 8% versus 5.4%.

Reason for the above-mentioned differences between NMR and crystal structures is not obvious, and we propose just a few possible explanations. Protein/DNA complexes form 65% of structures resolved by X-ray crystallography, but this fraction is only 17% in NMR. The higher number of protein/DNA complexes resolved by X-ray crystallography could perhaps explain a larger number of the A-form in crystal than in NMR structures as the A-form is often induced by interactions with proteins. A larger population of BI and a smaller population of BII in NMR structures cannot be explained so easily. Either of these forms has only limited sequence preferences, and there seem to be no obvious rationale supporting a hypothesis that crystal packing favors the BII over the BI conformation. A different hypothetical explanation could lie in the process of interpretation of the NMR experimental data. Their relative scarcity caused by the low density of protons, and sometimes equivocal interpretation of experiments such as indirect spin−spin couplings (“J-couplings”) may cause uncertainties especially in the assignment of torsions α and ζ of the phosphodiester linkage [116]. The resulting DNA structure may then be influenced by the refinement protocol in which the experimental restrains are combined with force fields in a computer simulation. Relatively low number of the experimental restraints and imperfection of the force fields, namely their incorrectly set torsion preferences, may perhaps favor BI over BII forms.

## Conclusions

In the present work we investigated several supervised machine-learning approaches (ridge regression (RR), multi-layer perceptron (MLP) neural network, radial basis function (RBF) neural network, and *k* nearest neighbors (*k*-NN)) to develop a protocol for an automatic classification of local DNA conformations. The classifiers were trained and tested using the previously published manually classified set of dinucleotides [32]. Various parameters of the machine learning methods were set to their optimum values utilizing a 10-fold cross-validation procedure. According to the results of our testing, the best method is *k*-nearest neighbor. This technique not only achieves high classification accuracy, but also allows identifying conformers that cannot be assigned to any of known classes. We subsequently investigated the unassigned conformers for the presence of new clusters using a modified clustering method based on the *leader algorithm*[89]. By the proposed machine learning workflow (Figure 1) we successfully analyzed X-ray and NMR structures of both naked and complexed DNA released until 15 February 2013. In addition to 18 conformational classes compiled in [32] we identified 6 new classes in X-ray structures, and no additional new classes in NMR data. We assigned four of these conformers to two structurally important DNA families: guanine quadruplexes and Holliday (four-way) junctions. The new clusters enhance structural annotation of *O. nova* telomeric G-quadruplex [32] and we were able to construct its consensus conformational map (Figure 3(d) and (e)). Comparison of frequencies of individual conformers found in X-ray and NMR structures showed that they have similar qualitative features so that DNA in the crystal phase and in solution populate the same regions of the conformational space. Observed differences between populations of X-ray and NMR conformers can be partially assigned to different composition of both datasets, partially to the refinement protocol of NMR structures that may favor BI over the BII form.

## Competing interests

The authors declare that they have no competing interests.

## Authors’ contributions

PČ designed and implemented MLP and RBF neural network models, performed tests of all used methods and drafted the manuscript. DS instigated the study, participated in its coordination, implemented the k-NN method, annotated new conformers, and drafted the manuscript. BS was responsible for data selection and data processing, performed the analyses of NMR data, and drafted the manuscript. JK implemented and applied the regularised regression method. JČ participated in data selection, data processing, and implemented additional support scripts. All authors read and approved the final manuscript.

## Supplementary Material

Additional file 1 **Data sets.***DatasetF* contains 4,567 dinucleotide “suite” units classified previously [32] into 18 classes. We used this classification as a “gold standard” in the present work. *DatasetF* was stratifically divided into the training set (sheet “DatasetF_train”) and into the test set (sheet “DatasetF_test”). Sheet “X-ray data” contains all crystallography data, and sheet “NMR data” contains all NMR data analyzed in the current work. Click here for file

Additional file 2 **Confusion matrices.** Confusion matrices of the ridge regression (RR), the multi-layer perceptron (MLP) neural network, the radial basis function (RBF) neural network and the *k* nearest neighbors (*k*-NN). The “true” class [32] is shown in the rows, and the class predicted by the given method is shown in the columns. Click here for file
